# Effect of Different Temperatures on Consumption of Two Spotted Mite, *Tetranychus urticae*, Eggs by the Predatory Thrips, *Scolothrips longicornis*

**DOI:** 10.1673/031.012.9801

**Published:** 2012-08-13

**Authors:** Hajar Pakyari, Annie Enkegaard

**Affiliations:** ^1^ Department of Plant Protection, Faculty of Agriculture, Islamic Azad University, Takestan Branch, Iran; ^2^ Aarhus University, Faculty of Science and Technology, Department of Agroecology, Research Centre Flakkebjerg, DK-4200 Slagelse, Denmark

**Keywords:** biological control, Thripidae, temperature threshold, Tetranychidae

## Abstract

Environmental variables such as temperature are important factors affecting the efficacy of biological control agents. This study evaluated the predation rate of the predatory thrips *Scolothrips longicornis* Priesner (Thysanoptera: Thripidae) against the two-spotted spider mite *Tetranychus urticae* Koch (Acari: Tetranychidae) under laboratory conditions. Based on daily and total prey consumption of different life stages of *S. longicornis* on spider mite eggs at temperatures covering the range suitable for development and survival of the predator (15° C to 37° C, 60 ± 10% RH, 16:8 L:D), there was a significant effect of temperature on prey consumption. The number of prey consumed daily by first and second instar larvae increased linearly with increasing temperature from 15 ^°^C to 37 ^°^C, whereas daily consumption of preovipositing and postovipositing females was uninfluenced by temperature. Lower temperature thresholds for consumption by first and second instar larvae of *S. longicornis* was estimated to be 6.8 ± 0.04° C and 4.6 ± 0.03° C, respectively. The daily consumption of ovipositing females followed a nonlinear pattern, with maximum daily predation estimated at 32.8° C. From the model used to describe consumption of ovipositing females, an upper threshold for consumption of 41.4° C was estimated. The performance of *S. longicornis* at the different temperatures is discussed in relation to its practical use in integrated pest control programs.

## Introduction

The two-spotted spider mite, *Tetranychus urticae* Koch (Acari: Tetranychidae), is a widespread agricultural pest, causing severe damage on a variety of greenhouse and field crops (Cranham 1985). Spider mites are difficult to control with pesticides (Nahar et al. 2005) due to inaccessibility of lower leaf surfaces, short life cycle, high reproductive capacity, and ability to develop resistance to miticides (Cranham and Helle 1985; Georghiou 1990).

Biological control, using natural enemies, is an alternative strategy to manage mites in agricultural systems. Natural enemies play a major role in the ecology of spider mites, including ladybird beetles (Coleoptera: Coccinellidae) (Obrycki and Kring 1998; Mori et al. 2005), predatory anthocorids (Heteroptera: Anthocoridae) (Coll and Ridgway 1995; Cocuzza et al. 1997), and predatory mites (Acari: Phytoseiidae) (Gotoh et al. 2004a; Friese and Gilstrap 1985). In addition, acarophagous thrips (Thysanoptera: Aeolothripidae, Thripidae) are important natural enemies, and have various degrees of specialization on various mites; however, all species of *Scolothrips* appear to be specialized on spider mites (Lewis 1973; Gilstrap and Oatman 1976). Several *Scolothrips* species have been shown to control spider mites, those species including *Scolothrips takahashii* Priesner (Yamasaki et al. 1983; Nakagawa 1993; Kishimoto 2003; Gotoh et al. 2004b), *Scolothrips sexmaculatus* (Pergande) (Mori 1967; Huffaker et al. 1970; McMurtry et al. 1970; Gilstrap and Oatman 1976), *Scolothrips indicus* Priesner (Ho and Chen 2001a, b), and *Scolothrips longicornis* Priesner. *S. longicornis* occurs in the Middle East, India, and North America (Priesner 1950; Gilstrap and Oatman 1976; Alavi and Kamali 1996). In Iran, this species is a commonly recorded predator of spider mites in outdoor bean, cucumber, and eggplant (Aydemir and Toros 1990; Pakyari et al. 2011a, b; Pakyari and Fathipour 2009). Some aspects of the biology of *S. longicornis* (functional response, mutual interference, life table characteristics, and feeding activity) have been studied (Gerlach and Sengonca 1986; Sengonca and Weigand 1988; Pakyari et al. 2009; Pakyari and Fathipour 2009), but the influence of temperature on prey consumption has not been examined to date.

As understanding of the effect of temperature on prey vs. natural enemy interactions may impact the success of biological control (Roy et al. 2002), the aim of this study was to determine the effect of different temperatures on daily and total prey consumption of *S. longicornis*. The results will help us to determine the potential of this predator for biological control against *T. urticae*.

## Materials and Methods

### Rearing of mites and thrips

A colony of two-spotted spider mites was initiated using individuals originally collected from cucumber fields (*Cucumis sativa* L. cv. Soltan) in the Varamin Tehran province. The mites were maintained on detached cucumber leaves, placed with the lower leaf surface facing up, on a layer of moist cotton inside 20 Petri dishes (150 mm in diameter). The lids of the Petri dishes had a 30 mm diameter hole covered with fine nylon mesh (150 µm) to allow for ventilation. The Petri dishes were kept in a climate chamber (Binder KBWS 240, Germany) at 26 ± 1° C, 60 ± 10% RH, and a 16:8 L:D photoperiod. A laboratory colony of *S. longicornis* was initiated using adults collected from the same cucumber fields. A single cucumber leaf was placed in a Petri dish (180 mm in diameter) as described above, and maintained in another climate chamber with similar conditions as above. Adult thrips were transferred to new, mite-infested cucumber leaves every 2 days. After a rearing period of 2 and 3 months for thrips and spider mites respectively, individuals were harvested from the colonies in order to be used for the experiments.

### Test arena

Leaf discs (30 mm in diameter) without major veins were excised from bean plants (*Phaseolous vulgaris* L. cv. Sunray) grown under laboratory conditions. Each disc was placed with the lower leaf surface facing up on a layer of moist cotton in a Petri dish (60 mm in diameter). The Petri dishes were ventilated through a nylon mesh-covered hole (15 mm in diameter) as described above.

### Experimental design

Laboratory experiments were conducted at six (± 1° C) temperatures (15° C, 20° C, 26° C, 30° C, 35° C and 37° C), 60 ± 10% RH, and 16:8 L:D photoperiod. The temperatures were chosen to cover the range suitable for development and survival of the predator (Pakyari et al. 2011a). The effect of temperature on prey consumption was determined for all feeding life stages (first and second instar larvae, adults) of *S. longicornis* by following cohorts of individuals. Cohorts were initiated by placing twenty mated adult females on bean leaf discs at 26° C for egg-laying. Sixty one-day-old eggs were subsequently kept in a climate chamber at each temperature until hatching, after which newly hatched first instar larvae were transferred individually to fresh leaf discs. Immature individuals were transferred to fresh leaf discs every 2 to 3 days until adult emergence, after which pairs of one male and one newly emerged female (maximum 1 day old) were placed in separate Petri dishes for mating. After 24 hours, males were removed, and the females were observed daily until death in order to record the onset and termination of oviposition. Females were transferred to fresh leaf discs every 2 to 3 days.

**Table 1.**
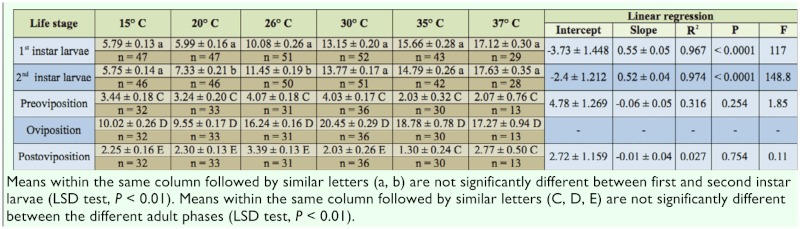
Daily prey (mean ± s.e.) consumption of eggs of *Tetranychus urticae* by different life stages of *Scolothrips longicornis* at six temperatures (±1° C) together with the parameters and statistics for the linear regressions of consumption versus temperature.

Throughout immature development and lifespan of females, thrips were daily fed a surplus of eggs of *T. urticae* (about 100 prey items offered daily for each larvae and female; the number of offered preys was determined from preliminary feeding experiments). The number of eggs consumed was counted daily under a stereomicroscope, after which the eggs were replenished. For the number of replicates, see Tables 1 and 2.

Immature development time, as well as the duration of the preoviposition, oviposition, and postoviposition phases, was determined for each temperature by daily inspection of the Petri dishes. The different larval instars were distinguished based on larval size, and on the presence of larval exuviae. The data on juvenile development and duration of the female phases were reported in Pakyari et al. (2011a) and Pakyari et al. (2011b), respectively, but are included here in order to be held in comparison with the results on consumption.

**Table 2.**
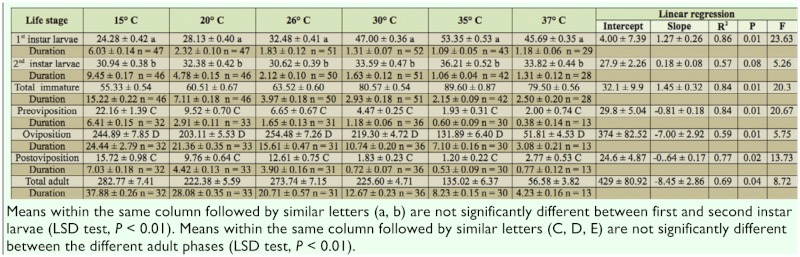
Total prey consumption (mean ± s.e.) on eggs of *Tetranychus urticae* and duration of the development phase (in days) of different life stages of *Scolothrips longicornis* at six temperatures (±1° C) together with the parameters and statistics for the linear regressions of consumption versus temperature, n = number of replicates for both consumption and duration.

### Statistical analysis

One-way analysis of variance (ANOVA) was performed to determine significance in prey consumption of *S. longicornis* among the different development stages using Minitab software (Minitab Inc. 2000). Significant differences were separated using multiple mean comparisons (LSD test (*P* < 0.05)). The relationship between consumption and temperature was analyzed with linear regression (using the SPSS statistical program (v. 13.0; SPSS 2004)), except in one case (daily consumption by ovipositing females) in which the nonlinear relationship was described by the following model (adapted from a model for description of temperature dependent development (Briere et al. 1999)):
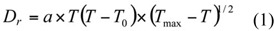

where *T* is the temperature, *a* is an empirical constant, *T*_0_ is the lower temperature threshold, and *T*_max_ is the higher temperature threshold for consumption. The nonlinear analysis was performed using the SPSS statistical program (v. 13.0; SPSS 2004).

## Results

Daily and total prey consumption by *S. longicornis* feeding on *T. urticae* eggs at the different temperatures is presented in Tables 1 and 2, respectively. There was a significant effect of temperature on daily and total consumption by *S. longicornis* first instar larvae, with daily consumption increasing linearly (P < 0.0001) from ∼ 6 to∼ 17 eggs/day, and with total consumption increasing linearly (P = 0.008) from ∼ 24 to ∼ 50 when temperature increased from 15° C to 37° C (Table 1). Temperature also had a significant influence on daily prey consumption (P < 0.0001), and an almost significant (P = 0.084) influence on total consumption of second instar. The number of prey consumed daily by second instars was generally of the same amount as for the first instar predators, except at 20° C and 26° C, temperatures at which second instars consumed more eggs (Table 1). Due to the generally longer development time (Table 2) of second instar larvae, total consumption by this stage was consistently higher at all temperatures compared with that of first instar larvae (Table 2). From the linear regression of daily consumption versus temperature, the lower temperature threshold (± s.e.) for consumption by first and secod instar larvae of *S. longicornis* was estimated to be 6.8 (± 0.04)° C and 4.6 (± 0.03)° C, respectively.

Female *S. longicornis* started consuming eggs the day after emergence at all temperatures. During oviposition, *S. longicornis* consumed the most eggs, with a maximum daily consumption of 20.45 eggs/day at 30° C (Table 1), and a maximum total consumption of 254.48 eggs at 26° C (Table 2). In both the preoviposition and postoviposition phasem the consumption was noticeably lower than during oviposition (Tables 1, 2).

The daily consumption in the preoviposition and postoviposition phase was uninfluenced by temperature (P > 0.254). Because temperature influenced the length of preoviposition and postoviposition (Table 2; Pakyari et al 2011a), total egg consumption in these phases decreased linearly (P < 0.02) from 22.16 and 15.72 eggs/day, respectively, at 15° C to 2.0 and 2.77 eggs/day, respectively, at 37° C.

In contrast, the daily consumption during oviposition followed a nonlinear pattern described (*R^2^* = 0.870) by (1) with the following parameter estimates (± s.e.): a = 0.0006 (± 0.003), *T_0_* = -3.4° C (± 12.0), *T_max_* = 41.4° C (± 2.6), and maximum daily predation estimated at 32.8° C.

The total consumption by females during the oviposition phase declined from 15° C to 20° C, followed by an increase from 20° C to 26° C, after which consumption steadily decreased as temperature increased (Table 2). The pattern reflects a combination of the linearly decreasing female oviposition period with increasing temperatures (Table 2, Pakyari et al. 2011b), and the nonlinear temperature dependent function for daily prey consumption. Overall the total consumption by ovipositing females could, however, be described by linear regression (P = 0.005) (Table 2).

## Discussion

The usefulness of a predator in the management of pests may relate, in part, to its capacity to perform adequately under a range of environmental conditions. This study determined the influence of temperature on consumption of spider mite eggs by *S. longicornis*, and has demonstrated a differential influence on the different life stages of the predator. Thus, daily consumption rate of the immature stages followed the same pattern for both first and second instar larvae, with the number of spider mite eggs consumed increasing linearly with temperature from 15° C to 37° C. However, preovipositing and postovipositing females were not affected by temperature, whereas the daily consumption of ovipositing females peaked at 32.8° C. The biological explanation for this difference in response to temperature between immature and female *S. longicornis* is not known. An increase in prey consumption by *Scolothrips* sp. with temperature up to 30° C has been demonstrated by others (Gerlach and Sengonca 1986; Lee et al. 1991), as well as a decrease in consumption with temperatures higher than 30° C (Gilstrap and Oatman 1976).

Daily consumption at 26° C by the immature stages of *S. longicornis* on eggs of *T. urticae* in the present study is similar to other studies on this predator, with daily consumption of 12.9 to 15.4 spider mite eggs at 25° C (Gerlach and Sengonca 1985; Gerlach and Sengonca 1986; Sengonca and Weigand 1988). Likewise, Gilstrap and Oatman (1976) found that immature *S. sexmaculatus* at 26° C consumed 11.7 spider mite eggs/day, although the prey in this case was *Tetranychus pacificus* McGregor (Acari :Tetranychidae).

Regarding the daily consumption by female *S. longicornis*, our results were significantly lower than reported by Gerlach and Sengonca (1986), who showed that female *S. longicornis* increased their predation on eggs of *T. cinnabarinus* Boisduval (Acari: Tetranychidae) from 58 to 64 as temperature increased from 15 to 35° C. Our results are also significantly lower than the daily consumption of *S. sexmaculatus* females for which Gilstrap and Oatman (1976) demonstrated, as they recorded an increase in predation on eggs of *T. pacificus* from 39 to 47 as temperature increased from 18 to 30° C. The lower consumption rate by females observed in our study may be due to differences in prey species, and in experimental conditions.

The higher daily egg consumption by ovipositing compared to preovipositing and postovipositing *S. longicornis* may be associated with additional food requirements for egg production.

The lower temperature threshold for consumption by immature *S. longicornis* was estimated to be about 4.6 to 6.8° C, which is lower than the previously reported values of lower temperature thresholds for development estimated for *S. takahashii* (approx. 13.3° C (Yamasaki et al. 1983; Gotoh et al. 2004b)) and for *S. sexmaculatus* (approx. 13.5° C (Gilstrap and Oatman 1976; Coville and Allen 1977)). The estimate of the lower temperature threshold for the consumption by ovipositing females has a large standard error, and can therefore not be taken into account as a proper estimate, whereas the estimated upper temperature threshold of 41.4° C is similar to that found for the development of *S. sexmaculatus* (40.6° C (Gilstrap and Oatman 1976)).

In comparison with other spider mite predators, the daily consumption of *T. urticae* eggs by female *S. longicornis* (24 eggs per day at 26° C) was higher than reported for female phytoseiid mites (Acari: Phytoseiidae) such as *Phytoseiulus persimilis* Athias-Henriot (14.9 eggs per day at 25° C) (Friese and Gilstrap 1985) and *Amblyseius californicus* (McGregor) (13.4 eggs per day at 25° C) (Gotoh et al. 2004a). However, consumption was notably lower than reported for larger and more voracious predators, e.g. female *Stethorus punctillum* Weise (Coleoptera: Coccinellidae, Heteroptera: Anthocoridae) (60–80 eggs per day at 30° C) (Parvin et al. 2010), and female *Macrolophus caliginosus* Wagner (Heteroptera: Miridae) (about 100 eggs per day at 22° C) (Enkegaard et al. 2001). However, compared to phytoseiid mites with well-documented control capability towards spider mites (e.g. Gerlach and Sengonca 1985; Zhang and Croft 1994; Kazak 2008), the predation capacity of *S. longicornis* demonstrated here holds good promises for the exploitation of this predatory thrips for control of *T. urticae* in crops where the temperature range between 20° C and 30–35° C, as is the case, for example, in Mediterranean greenhouses. Thus, although the innate capacity for increase of *S. longicornis* has been documented (Pakyari et al. 2011b) to be generally lower than that of phytoseiid mites (e.g. Sabelis 1985, 1991), the higher predation capacity may compensate for this. Further studies on, for example, the influence of humidity and of different prey ratios (eggs, active stages) on the predation capacity of *S. longicornis* will be needed to further nuance the evaluation of this predator as a biocontrol agent of spider mites.
